# The German Society of Naturalists and Physicians

**Published:** 1888-02

**Authors:** 


### THE GERMAN SOCIETY OF NATURALISTS AND PHYSICIANS.

Natural Science in Schools. — At the annual meeting of the German Society of Naturalists and Physicians, Wiesebaden, September, 1887, Herr Preyer (Wilhelm) read a paper on Natural Science and Schools. He maintained that (1) the laws of biology are violated by the forced intellectual development aimed at in schools as at present conducted; and (2) that the study of Latin and Greek is unnecessary and unprofitable, takes the place of objective and useful studies—for example, the natural sciences—and is altogether a painful infliction upon the young. The idea that modern culture rests upon a historic continuity with the classical ages, is based upon a self-deception; that continuity has been already broken by Luther, by Galileo, by Copernicus (and he might have added, by Isaac Newton). "The armies of Germany, not book-learning, have made her great." (!) Herr Preyer is on safe ground when he dilates on the injurious effects of school life in Germany upon the development of the body as a whole. Its harmonious growth is retarded and deranged by the artificial restrictions, the want of exercise, the over-studying (more than half the students are more or less short-sighted), and various other injurious influences. We are more fortunate in this respect in England, at least as regards outdoor exercise and medical supervision, but the natural sciences are still almost ignored.

Origin of Species.—Professor Virchow next delivered an address dealing with the Darwinian theory. Darwin released the world from Cuvier's dogma of the unalterability of species ; the species as a real object does not exist ; only the individual exists. The individual is made up of organs. Now, if the species were unalterable, then individuals, like the separate parts of a crystal, would exactly resemble each other. But no one demands this, and, in fact, only the average of the special attributes of various individuals is required to characterize the species. The individuals are the more variable, as the number of their organs is greater. The variability of the individual is the foundation of the variability of the species. Virchow is not antagonistic to the theory of Darwin, but only to the excursive tendency of his disciples, especially as regards the acquirement and heredity of variations. Referring to the origin of life, the doctrine that it has always existed on the globe is pronounced false. There was once a time when nothing living existed. The Bible account of the creation, and the theory of primitive generation and evolution, were opposed to each other. The latter was only a presumption, a claim, of natural science. The sources of such generation were unknown to us, and Pasteur had refuted the idea that organic matter could arise from an agitation of inorganic matter. Darwin's idea was, nevertheless, a highly fruitful mental conception, an energetic ferment in the scientific world. Both Haeckel and Vogt had allowed that life might have originated on the earth in more ways than one. As regards the origin of man, the first man could only have existed towards the end of the Tertiary period, or the beginning of the diluvium deposits, for no remains of man had been found in earlier formations. A few flints, bearing evidence of being shaped, were all we had of man even in the Tertiary series. It was not till the alluvial deposits were reached that human bones were found, and these were not so numerous as was supposed. The skull from the Neander valley, and the lower jaw from the Schipka cavern, gave no distinct evidence of a lower grade of organization than the present. The (evolutionists) "fanatics" rejoiced in being able to assert that these remains indicated a man corresponding to a living Australian native, but the latter, after all, was neither an ape nor a pro-anthropoidal man. The question of the origin of man was, in fact, an insoluble problem. In regard to the formation of the human race, the theologians and the natural science enthusiasts (*Fanatiker*) were of one mind; they both pre-supposed a primitive pair of individuals.

PLANT-LIFE AND PLANT-RESPIRATION.—Professor Detmer, of Jena, gave an address on Plant-life and Plant-respiration, and showed that many vital processes are common to animals and plants—for example, respiration (intra-molecular), digestion, and even the development of heat. The professor pleaded earnestly for an extension of natural-science teaching as advocated by Preyer. Such teaching did not have a materialist tendency, as generally supposed; on the contrary, it tended towards the ideal, if more weight was laid upon the vast connection subsisting between natural phenomena than upon experimental details.

PUTREFACTION AND INFECTIOUS DISEASES.—The address by Dr. F. Hueppe, of Wiesbaden, was perhaps the most interesting for the medical world. The title was: On the Relations between Putrefaction and Infectious Diseases, and the author began by a pithy and lucid historical sketch, extending from Hippocrates (of course) to the present time. In the course of this sketch we are reminded that Pringle and J. P. Frank, in the eighteenth century, declared that "putrid fevers" were due to putrefactions going on external to the organism, and Pringle recognized a relationship between epidemics of such fevers and the depth of the water level below the surface of the ground. Previously to this, if we except Frascatori in the Middle Ages, scarcely any attention had been directed to external influences from the time of Hippocrates, and, after him, of Diodorus. As for putrefaction itself, the idea that this was caused by the growth of low organisms was never even broached till the present century. The old view was that the term "life," connoted the absence of putrefaction, apparently because the healthy body resisted invasion by pathogenic germs. Against this view Henle strongly set himself, taking for his basis the investigations of Schwann and Cagniard Latour as regards *infusoria*, and those of Bassi and Audouin on infectious diseases. In fact, Henle's ideas were very advanced, for he thought that infective diseases were due to germs starting from without, and that these germs were due to putrefaction. Further, Henle ventured to argue that "it depends on special conditions what kinds of *infusoria* and plants develop, and the different kinds are not equally inimical to health." The natural development from this is that the living tissues resist pathogenic organisms during health, but that if this resistance be overcome the invading organisms develop, and decomposition goes on within the body, much as in external putrefaction, only modified in form and derivation by the tissues; and this conclusion has been confirmed by the investigations into the chemistry of bacteria by Nencki, Gautier, Selmi, and, above all, Brieger. Pettenkofer made a further step forward, for he divided the still hypothetical "infectious material" into entogenous and ectogenous. But his conceptions were too absolute. Pettenkofer held that the germs of infectious diseases were discharges from the body in an unfit, ineffective condition, and that only after a process of ripening outside the body could they again produce the original disease. We know that the case is otherwise; that, as a rule, such germs are most effective when discharged from the body, and that they do not acquire increased vigor from contact with mother earth, as certain heroes of old did, but rather tend to lose vigor and gradually perish. Nevertheless, we owe Pettenkofer a great debt for so early recognizing that many disease-organisms do go through certain stages of development outside the body—in some cases, as in the spore-formation of anthrax bacilli, only outside it. Our knowledge now begins to be more precise regarding disease germs. Pasteur definitely established the fact that putrefaction and fermentation processes are due to the growth of the respective organisms. Next, as regards disease, F. Cohn put all the disease-producing organisms in one class apart. Davaine had already shown that anthrax bacilli were excitants of ordinary septicæmia. Koch taught us the isolation and cultivation of bacteri, and Panum showed that putrid fluids may act as poisons even when no organisms are present. By this last step, previous pathological conceptions were etiologically fixed, and "putrid intoxications," due to poisons elaborated by bacteria, were distinguished by diseases caused by the entry and development of the specific bacteria themselves within the system. It was afterwards shown that the system could be intoxicated from within by bacteria. To go back for a moment to the beginning of our own century, Majendie's researches deserve mention here, because he showed that certain substances were developed in septicæmias (in animals), which, when transferred to other animals, set up a similar disease. Semmelweiss, "the well-known gifted founder of aseptic surgery," extended this observation to man, and by his success in the treatment of puerperal fever was the first to establish a beneficient prophylactic therapeutical system. It will be seen that many problems regarding disease-producing organisms were only rendered possible of solution through the attempts of Pasteur, Hallier, and Klebs—acting with ideas contrary to those of Cohn, who placed all such organisms in one class—to cultivate and separate the different kinds of bacteria, attempts which Koch carried on to a glorious frution. It is now known, as regards a whole series of pathogenic bacteria, that they possess a saprophytic stage, and that their "parasitism" in the human body is not at all necessary to their existence as species, but only accidental, a view which Henle and Pettenkofer from epidemiological considerations had already strenuously advocated. Such is the substance of the historical sketch given in this remarkable address; the rest of which deals with recent systems of classification of bacteria, and of the diseases arising from them, a large part being devoted to the various organs of the body, as they are mainly involved in different diseases, especially cholera nostras, and cholera Asiatica. As regards this part, we have no space for further representations of the author's views, but some concluding remarks may be given as reflecting creditably on hygienic medicine in this country: "As long as a circulation of material goes on, as long as putrefactive processes are necessary as affording an intermediate stage between animal and plant life, so long will micro-organisms exist, adapted for the destruction of albumen, or by an alteration of the processes for albumen putrefaction. And so long as this occurs, organisms will continue to exist, which, owing to this adaptation, are capable of setting up disease in the human body. We can never altogether conquer putrefaction — *tamen usque recurret.* But if — to quote Lord Palmerston — we regard dirt as only something in the wrong place, and regard putrefactive processes in our immediate neighborhood as in the wrong place, because uncontrolable and dangerous, we are then able to fight with those processes and their dangers without sacrificing the necessary processes for the circulation of material, together with their advantages. * * * Let these processes go on in the *humus* of the garden and the field, the meadow and the forest, where proper adaptation for plant life exist. * * * I adopt the English conception, which regards cleanliness — not a mere facade cleanliness, though—as health itself, and the best means of fighting against infectious diseases."

